# Supplementary material for: Biodegradation of Polyethylene by Enterobacter sp. D1 from the Guts of Wax Moth Galleria mellonella
Source: Int J Environ Res Public Health. 2019 May 31;16(11):1941. doi: 10.3390/ijerph16111941 (PMC6604253; doi:10.3390/ijerph16111941)
Supplement: Supplementary file 1 [file ijerph-16-01941-s001.pdf]

International Journal of
Environmental Research and Public Health

Supplementary Material

Biodegradation of Polyethylene by *Enterobacter* sp. D1 from the Guts of Wax Moth *Galleria Mellonella*

Liu Ren, Lina Men, Zhiwei Zhang, Feifei Guan, Jian Tian, Bin Wang, Jihua Wang *, Yuhong Zhang * and Wei Zhang [2]

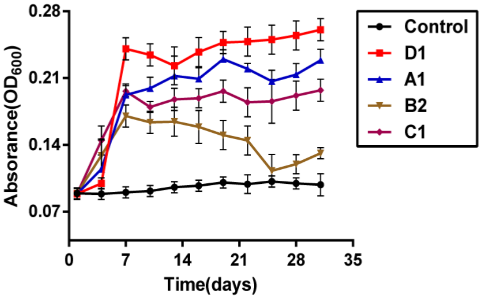

Figure S1. The changes of OD_{600} of the 4 strains during the 31-day cultivation. The 4 strians were screened from the gut homogenate of the wax moth that could grow in the LPEM containing 5% PE.

Table S1. The estimated OD_{600} of the every sample during the 31-day cultivation.

Days / *Sample*	*1*	*4*	*7*	*10*	*13*	*16*	*19*	*22*	*25*	*28*	*31*
	**0.089**	**0.089**	**0.09**	**0.092**	**0.096**	**0.097**	**0.101**	**0.1**	**0.102**	**0.1**	**0.099**
Control	**0.089**	**0.089**	**0.09**	**0.092**	**0.095**	**0.098**	**0.1**	**0.099**	**0.102**	**0.1**	**0.099**
	**0.09**	**0.088**	**0.091**	**0.091**	**0.096**	**0.097**	**0.101**	**0.098**	**0.101**	**0.099**	**0.097**
	**0.089**	**0.1**	**0.24**	**0.235**	**0.225**	**0.239**	**0.248**	**0.248**	**0.25**	**0.253**	**0.26**
D1	**0.088**	**0.099**	**0.24**	**0.235**	**0.221**	**0.236**	**0.248**	**0.247**	**0.252**	**0.256**	**0.262**
	**0.089**	**0.1**	**0.242**	**0.233**	**0.223**	**0.237**	**0.246**	**0.25**	**0.249**	**0.255**	**0.26**
	**0.089**	**0.112**	**0.193**	**0.2**	**0.213**	**0.21**	**0.231**	**0.22**	**0.208**	**0.215**	**0.23**
A1	**0.088**	**0.114**	**0.191**	**0.2**	**0.21**	**0.208**	**0.229**	**0.219**	**0.206**	**0.213**	**0.228**
	**0.089**	**0.118**	**0.193**	**0.198**	**0.214**	**0.21**	**0.23**	**0.22**	**0.206**	**0.213**	**0.228**
	**0.089**	**0.127**	**0.171**	**0.165**	**0.163**	**0.157**	**0.149**	**0.146**	**0.114**	**0.12**	**0.131**
B2	**0.089**	**0.13**	**0.169**	**0.163**	**0.164**	**0.16**	**0.152**	**0.143**	**0.114**	**0.119**	**0.132**
	**0.09**	**0.13**	**0.171**	**0.163**	**0.166**	**0.16**	**0.15**	**0.145**	**0.111**	**0.121**	**0.131**
	**0.089**	**0.145**	**0.197**	**0.18**	**0.189**	**0.19**	**0.197**	**0.186**	**0.184**	**0.19**	**0.197**
C1	**0.088**	**0.146**	**0.193**	**0.179**	**0.187**	**0.189**	**0.195**	**0.183**	**0.186**	**0.192**	**0.196**
	**0.089**	**0.143**	**0.199**	**0.18**	**0.187**	**0.188**	**0.197**	**0.185**	**0.187**	**0.193**	**0.199**

Table S2. Statistical summary of the atom C and O on the surface of PE film.

	D1		*Control*	
Atom	**C**	**O**	**C**	**O**
% Atom	**96.78**	**3.22**	**98.88**	**1.12**
% Atom	**96.84**	**3.16**	**98.99**	**1.01**
% Atom	**96.98**	**3.02**	**98.67**	**1.33**
Mean	**96.8667**	**3.1333**	**98.8467**	**1.1533**
SD	**.10263**	**.10263**	**.16258**	**.16258**

Table S3. Independent samples test for the atomic percentage to the every sample.

		t	*df*	*Sig. (2-tailed)*	*Mean Difference*	*Std. Error Difference*	*95% Confidence Interval of the Difference*	
							Lower	*Upper*
% C	*Equal variances assumed*	-17.837	4	.000	-1.98000	.11101	-2.28820	-1.67180
	Equal variances not assumed	-17.837	3.376	.000	-1.98000	.11101	-2.31204	-1.64796
% O	*Equal variances assumed*	-17.837	4	.000	1.98000	.11101	1.67180	2.28820
	Equal variances not assumed	-17.837	3.376	.000	1.98000	.11101	1.64796	2.31204

Table S4. Detection of the significantly increased compounds from the bacterial solution of the D1 group compared with the control group by LC-MS at the end of 31-day cultivation.

Metabolite name	*Average Rt(min)*	*Abundance(Control)*	*Abundance(D1)*
6-Methyl-5-hepten-2-ol	**0.93**	**3825**	**13831**
D-(-)-Erythrose	**1.29**	**3424**	**41176**
Cystine	**1.3**	**585**	**33337**
N-ACETYLGLYCINE	**1.35**	**399**	**11056**
5-Aminovaleric acid	**1.35**	**710**	**16524**
Ala-Val	**1.51**	**370**	**13702**
Monobenzyl phthalate	**4.24**	**79**	**19480**
N-Acetylglutamic acid	**4.3**	**10**	**3996**
Pantothenic acid	**5.25**	**30**	**27658**
Proline-hydroxyproline	**6.37**	**50**	**6827**
Glu-Thr	**6.38**	**57**	**8292**
Phosphatidylethanolamine 22	**16.92**	**13604**	**46191**
PG(18:1/10-HDoHE)	**17.28**	**2511**	**14148**
Ethyldodecanoate	**16.97**	**5857**	**21591**
1-(3,4-dimethoxyphenyl)-2-[4-[5-[4-[1-(3,4-dimethoxyphenyl)-1-hydroxypropan-2-yl]oxy-3-methoxyphenyl]-3,4-dimethyl oxolan-2-yl]-2-methoxyphenoxy]propan-	**15.82**	**68**	**16018**

1-ol			
(2R,3R,4S,5S,6R)-2-hexoxy-6-(hydroxymethyl)oxane-3,4,5-triol	**16.53**	**9803**	**21784**
4-[(2S)-2-hydroxy-3-methyl-3-[(2S,3R,4S,5S,6R)-3,4,5-trihydroxy-6-(hydroxymethyl)oxan-2-yl]oxybutoxy]furo[3,2-g]chromen-7-one	**16.87**	**6190**	**12208**
Methyl-13-hydroperoxy-delta9E,11E-octadecadienoic acid	**17.18**	**25951**	**51852**
(2S)-3-(3,4-dihydroxyphenyl)-2-[[(E)-3-(3,4-dihydroxyphenyl)prop-2-enoyl]amino]propanoic acid	**1.49**	**828**	**6828**

© 2019 by the authors. Submitted for possible open access publication under the terms and conditions of the Creative Commons Attribution (CC BY) license (http://creativecommons.org/licenses/by/4.0/).
